# Enhancing Well-Being: Optimizing Service Delivery in Neighbourhood Mental Health Team (NMHT) for Administrative Staff and Service users

**DOI:** 10.1192/bjo.2024.338

**Published:** 2024-08-01

**Authors:** Harith Ali, Danica Moller, Amra Selim, Taz Meah, Selma Ali

**Affiliations:** East London Foundation Trust, London, United Kingdom

## Abstract

**Aims:**

Promoting the well-being of staff is paramount within mental health services. However, a common issue arises where administrative personnel, often serving as the primary point of contact for service users, engage in mental health-related interactions without formal training. This deficiency can adversely affect their well-being, leading to diminished team morale and increased staff turnover, consequently impacting the quality of care provided by the Neighborhood Mental Health Team (NMHT). Moreover, it can contribute to dissatisfaction among service users, jeopardizing their rapport with the service. We aim to improve the wellbeing of staff and service users and to optimize service delivery at the local NMHT.

**Methods:**

Data were gathered from a local NMHT catering to 1200 service users in the borough of Tower Hamlets in London. A pre- and post-implementation questionnaire was administered to both service users and six administrative staff members. The questionnaire highlighted several areas for improvement, including a lack of mental health understanding among administrative staff, reported low confidence when handling certain phone inquiries, and service user complaints. Change initiatives were then devised to address these concerns and evaluate their impact on enhancing the experience for both service users and administrative staff.

**Results:**

Administrative staff uniformly expressed the need for increased mental health training prior to commencing their roles. Implementation of targeted change initiatives led to noticeable improvements in service user satisfaction and staff confidence in managing phone interactions. These enhancements culminated in an overall advancement in service delivery.

**Conclusion:**

Through the strategic implementation of change initiatives informed by our initial findings, we not only augmented mental health literacy among administrative staff and service users but also bolstered their well-being. Consequently, this directly translated into an amelioration of local service offerings. Further research is warranted to ascertain the long-term efficacy of these innovative interventions.

